# New Classes of Alanine Racemase Inhibitors Identified by High-Throughput Screening Show Antimicrobial Activity against *Mycobacterium tuberculosis*

**DOI:** 10.1371/journal.pone.0020374

**Published:** 2011-05-26

**Authors:** Karen G. Anthony, Ulrich Strych, Kacheong R. Yeung, Carolyn S. Shoen, Oriana Perez, Kurt L. Krause, Michael H. Cynamon, Paul A. Aristoff, Raymond A. Koski

**Affiliations:** 1 L2 Diagnostics, LLC, New Haven, Connecticut, United States of America; 2 Department of Biology and Biochemistry, University of Houston, Houston, Texas, United States of America; 3 Veterans Affairs Medical Center, Syracuse, New York, United States of America; 4 Department of Biochemistry, University of Otago, Dunedin, New Zealand; University of Hyderabad, India

## Abstract

**Background:**

In an effort to discover new drugs to treat tuberculosis (TB) we chose alanine racemase as the target of our drug discovery efforts. In *Mycobacterium tuberculosis*, the causative agent of TB, alanine racemase plays an essential role in cell wall synthesis as it racemizes L-alanine into D-alanine, a key building block in the biosynthesis of peptidoglycan. Good antimicrobial effects have been achieved by inhibition of this enzyme with suicide substrates, but the clinical utility of this class of inhibitors is limited due to their lack of target specificity and toxicity. Therefore, inhibitors that are not substrate analogs and that act through different mechanisms of enzyme inhibition are necessary for therapeutic development for this drug target.

**Methodology/Principal Findings:**

To obtain non-substrate alanine racemase inhibitors, we developed a high-throughput screening platform and screened 53,000 small molecule compounds for enzyme-specific inhibitors. We examined the ‘hits’ for structural novelty, antimicrobial activity against *M. tuberculosis*, general cellular cytotoxicity, and mechanism of enzyme inhibition. We identified seventeen novel non-substrate alanine racemase inhibitors that are structurally different than any currently known enzyme inhibitors. Seven of these are active against *M. tuberculosis* and minimally cytotoxic against mammalian cells.

**Conclusions/Significance:**

This study highlights the feasibility of obtaining novel alanine racemase inhibitor lead compounds by high-throughput screening for development of new anti-TB agents.

## Introduction

Discovery and development of effective chemotherapeutic agents for TB, a disease that affects one third of the world's population and kills 1–2 million people a year, is a top health priority [1], [2]. This need is further underscored by the lethal synergy of TB and HIV epidemics, and the emergence of multiple and extensively drug resistant (MDR and XDR) forms of the disease that are difficult to treat with the existing drug regimen [3], [4], [5], [6], [7], [8]. Despite this need, no new drug classes have been specifically marketed for TB in the last forty years [9], in part owing to a lengthy and costly process that takes almost two decades for drug approval [10]. One strategy that will allow for a rapid path to new anti-TB agents is to discover new classes of compounds against already validated drug targets.

In *M. tuberculosis*, one such target is alanine racemase (EC 5.1.1.1), a pyridoxal 5′-phosphate (PLP)-containing enzyme required for bacterial growth [11], [12], [13], [14]. This enzyme catalyzes the racemization of L-alanine to D-alanine, which is an essential precursor for the pentapeptide cross-bridge of the peptidoglycan layer in bacterial cell wall [15], [16].

Due to its essential nature, coupled with the absence of a human homolog, alanine racemase has long been an attractive drug target. We have previously reported the crystal structure of the *M. tuberculosis* alanine racemase [17]. The active form of the enzyme is an obligatory dimer containing two monomers of 43 kDa in head-to-tail orientation. Residues from both monomers contribute to the two active sites, where PLP and alanine bind.

**Figure 1:**
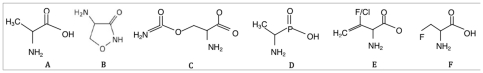
Substrate and selected inhibitors of alanine racemase. (A) alanine, (B) D-cycloserine, (C) o-carbamyl-D-serine, (D) L-alanine phosphonic acid, (E) fluoro- or chloro-vinyl glycine, and (F) fluoro-alanine.

Most known enzyme inhibitors bind solely to the substrate-binding region proximal to PLP. Shown in Figure 1, are several well-known alanine racemase inhibitors. Cycloserine and o-carbamyl-D-serine are two natural antibiotics known to inhibit alanine racemase [18], [19]. Only cycloserine has been developed commercially for the treatment of TB, but its clinical utility is limited due to toxicity issues arising from lack of target-specificity [20]. By virtue of its primary amine, cycloserine inactivates alanine racemase by engaging the enzyme-bound co-factor. Since PLP-dependent enzymes are ubiquitous in nature, cycloserine is not target-specific. Attempts to improve the activity or specificity through alteration of the cycloserine ring of side chains have not, to date, been successful [21], [22]. Additional alanine racemase inhibitors include β,β,β-trifluoroalanine, alanine phosphonate [23], 1-amino-cyclopropane phosphonate [24] and β-chloro- and β-fluoroalanine [25]. Like cycloserine, all of these inhibitors are alanine analogs that contain primary amines, and as such, likely will inhibit other PLP-dependent enzymes [26], [27], [28]. Therefore, alanine racemase inhibitors that are not substrate analogs and lacking primary amines in their structures are necessary for therapeutic drug development.

Structure-guided drug design has been employed to identify novel alanine racemase inhibitors [17], [29], [30], [31]. Small molecules in the 200–350 MW range have been successfully docked to the active site [17], [31]. These efforts, however, have not yet produced strong inhibitors with antimicrobial activity against the TB bacterium. The availability of a convenient alanine racemase assay that is amenable for high-throughput screening (HTS) has made it possible to screen for enzyme inhibitors. The overall aim of this study was to optimize and utilize the alanine racemase assay for HTS in search of novel enzyme inhibitors. Here, we report the identification of several novel classes of alanine racemase inhibitors that are not substrate analogs. Several of these inhibitors are active against *M. tuberculosis* and show limited cytotoxicity against mammalian cells. This study thus highlights the feasibility of HTS as a rapid and effective approach to obtain novel alanine racemase inhibitors for development as anti-TB agents.

## Materials and Methods

### Reagents

D-alanine, L-alanine, L-alanine dehydrogenase (*Bacillus subtilis*), and β-NAD-sodium salt were purchased from Sigma Aldrich (St. Louis, MO). Tricine and Tris were purchased from American Bioanalytical. Recombinant *M. tuberculosis* alanine racemase was expressed in *E. coli* as an N-terminal polyhistidine fusion. The cloning, expression and purification of this recombinant enzyme have been previously described [32].

### Alanine racemase and L-alanine dehydrogenase assay adaptation to 384-well format

The coupled alanine racemase assay of Esaki and Walsh [33], which measures the racemization of D- to L-alanine, was adapted to 384-well plate format. The assay was modified by varying the concentrations of alanine racemase, D-alanine, NAD, and L-alanine dehydrogenase in a Tricine buffer (100 mM, pH 8.5). The optimized HTS assay reaction mixture consisted of 12 nM alanine racemase, 1 mM NAD, 0.03 units/ml L-alanine dehydrogenase, and 2.5 mM D-alanine in 100 mM Tris-Tricine. Forty microliters of this reaction mixture were added to each well of a 384-well plate (Corning 3710). After a 15-minute incubation, fluorescence intensity associated with NADH, produced during the conversion of the racemized alanine to pyruvate, was measured in an EnVision plate reader (PerkinElmer, Waltham, MA) with excitation/emission at 340/460 nm. A reaction cocktail without the D-alanine substrate was used as the background control.

Assay components to measure the L-alanine dehydrogenase coupling enzyme activity consisted of L-alanine, NAD, L-alanine dehydrogenase and L-alanine in a Tris-Tricine buffer (100 mM, pH 8.5). Concentrations of these components were varied following the assay procedure described for the alanine racemase assay. The HTS assay conditions consisted of 1 mM NAD, 0.01 units/ml L-alanine dehydrogenase, and 0.1 mM L-alanine in 100 mM Tris-Tricine buffer.

### HTS procedures

Thirty microliters of the reaction cocktail containing 12 nM alanine racemase, 1 mM NAD, 0.03 units/ml L-alanine dehydrogenase in 100 mM Tris-Tricine buffer, were transferred to each well of a 384-well plate using an automated liquid dispenser (WellMate, Matrix Technologies Corp., Hudson, NH). The plates were loaded onto a pin transfer robot (Epson America Inc., Long Beach, CA) and 0.1 µl of test substance (5 mg/ml stock for small molecule compounds or 15 mg/ml natural product extracts) from a master library plate was transferred to the test wells. After a 30-minute incubation, 10 µl of 10 mM D-alanine (test and positive control wells) or 10 µl of water (negative control wells) were added to each well. NADH fluorescence intensity was measured in the EnVision plate reader after incubation for 20 minutes.

The HTS procedure for the L-alanine dehydrogenase assay was essentially as described for the alanine racemase assay. The reaction cocktail containing 1 mM NAD and 0.03 units/ml L-alanine dehydrogenase in 100 mM Tris-Tricine buffer was incubated with 0.1 µl each test substance for 30 minutes. Enzyme reactions were initiated by adding 10 µl of either 0.4 mM L-alanine (test and positive control wells) or 10 µl of water (negative control wells), and the reaction was read as described for the alanine racemase assay.

Statistical significance of a positive signal was determined by calculating the Z′-factor for each plate as described in [34], using the following formula: Z′ = 1−[3(SD signal+SD background)/(M signal−M background)], where signal is the negative control and background is the positive control, SD is the standard deviation, and M is the mean.

### Compound libraries

High-throughput screening was performed at the National Screening Laboratory for Regional Centers of Excellence for Biodefense and Emerging Infectious Diseases Research (NSRB) at Harvard Medical School. The libraries of 53,000 small molecules screened included therapeutic compounds approved by FDA, purified natural products extracts, and compounds purchased from BioMol TimTec (Plymouth Meeting, PA), Prestwick (Ilkirch, France), ChemBridge (San Diego, CA), ENAMINE (Kiev, Ukraine), Maybridge (Cornwall, UK), ChemDiv (San Diego, CA), and MicroSource Diversity System's NINDS custom collection (Gaylordsville, CT) as well as collections from the National Cancer Institute and Harvard Medical School.

### Enzyme IC_50_ determination

The HTS assay method was used for IC_50_ determination. Five-fold dilution series of compounds (in DMSO) were prepared, and added to 30 µl of reaction cocktail in 384-well plates to yield final inhibitor concentrations of 5.7 µg/ml, 1.1 µg/ml, 229 ng/ml, 46 ng/ml, and 9 ng/ml. Each concentration was tested in triplicate. After a 30-minute incubation, 10 µl of substrate was added and fluorescence intensity was measured after a 20-minute incubation. Percent inhibition at each inhibitor concentration was calculated with respect to a negative control with no inhibitor. The results were fitted onto a sigmoidal dose-response curve using Prism software (GraphPad Software Inc., La Jolla, CA) to calculate the IC_50_ (compound concentration that causes 50% inhibition).

### Antimicrobial susceptibility studies against *M. tuberculosis*

Antimicrobial activities of compounds were initially tested in the BACTEC system (BD, Franklin Lakes, NJ) with MGIT 960 incubator/detector and BBL MGIT *Mycobacteria* Growth Indicator tubes. Duplicate tubes were inoculated with a 1∶500 dilution of *M. tuberculosis* H37Rv suspension (turbidity of 0.5 McFarland standard). The inhibitors, dissolved in dimethylsulfoxide (DMSO), were added to each tube to a final concentration of 50 and 100 µg/ml. Appropriate controls included cycloserine as a positive control for growth inhibition (50 and 100 µg/ml), and DMSO solvent. The tubes were placed in the BACTEC MGIT 960 system and incubated at 37°C. Growth was monitored hourly for 42 days. In the BACTEC system, oxygen consumption by actively respiring bacteria is detected as an increase in fluorescence of an oxygen-quenched indicator, and no change in fluorescence intensity between the first and the last day of the experiment indicates growth inhibition.

Compounds that showed activity in the BACTEC system were re-evaluated using the microbroth dilution method to determine the minimal inhibitory concentrations (MICs) as we have previously reported [35]. Ninety-six-well microtiter plates (Corning) were filled with 7H10 broth, pH 6.6. Six serial two-fold dilutions of inhibitors (to yield final concentrations of 25, 12.5, 6.25, 3.125, 1.56 and 0.78 µg/ml) were added to appropriate wells to which 5×10^4^ viable *M. tuberculosis* H37Rv were added. The plates were incubated at 37°C and growth was visually inspected after 21 days. The MIC was defined as the lowest concentration of compound that did not yield visible turbidity. To determine the effect of D-alanine, MIC experiments were performed with 5 mM D-alanine in the growth medium.

### Compound cytoxicity studies

HeLa cells were grown to confluence overnight (37°C, 5% CO_2_) in Dulbecco's Modified Eagle Medium with 10% fetal bovine serum (Invitrogen, Carlsbad, CA). The following day, 3000 cells were plated into each well of a 96-well plate and grown overnight. Compounds were diluted in culture medium and added to the cells to yield final concentrations of 100, 50, 25, 12.5, and 6.25 µg/ml. The cells were exposed to the compounds for 48 hours, after which the effect on cells was determined using the WST-1 Cell Proliferation Reagent (Roche Applied Science, Indianapolis, IN) according to manufacturer's protocols. Absorbance at 450 nm was used to calculate percent cytotoxicity with respect to a DMSO solvent control. The results were fitted onto a sigmoidal dose-response curve using Prism software to calculate the TC_50_ (compound concentration that causes 50% cell death).

### *K_i_* determination

For *Ki* determination, alanine racemase was incubated with varying concentrations of inhibitors (0.98, 0.49, 0.43, 0.38, 0.31, 0.30, 0.29, 0.25, 0.17, 0.15, 0.10, 0.09, 0.08, 0.05, 0.01 mM). The mixtures were aliquoted and varying concentrations of D-alanine (2.5, 2, 1.5, 1, and 0.5 mM) in the reaction cocktail were added. After a 10-minute incubation, absorbance of NADH at 340 nm was read and enzyme velocity plots were generated. For each inhibitor concentration, the reciprocal of the enzyme reaction velocity versus the reciprocal of the substrate concentration was plotted in a Lineweaver-Burk plot. Subsequently, in a secondary plot, the slope at each inhibitor concentration (y = K_m[inhibitor]_/V_[inhibitor]_) was plotted versus the concentration of the inhibitor (X-axis). The data were fitted to a linear regression, and *Ki* was determined from the intercept on the inhibitor axis. All assays were performed in triplicate.

### Electrospray ionizaton mass spectrometry

Electrospray ionization mass spectrometry (ESMS) was performed to detect direct interactions of inhibitors with enzyme. A mixture of 15 µl of sample (4 µM alanine racemase with 1 mM inhibitors) was diluted with 15 µl 2% acetonitrile/0.1% formic acid. Samples were then de-salted using C4 ZipTip, eluted into 15 µl 60% acetonitrile/0.1% formic acid, and directly injected into Qtof-micro MS instrument (Waters Corp., Milford, MA).

## Results

### Development of HTS assays to screen for *M. tuberculosis* alanine racemase inhibitors

**Figure 2:**
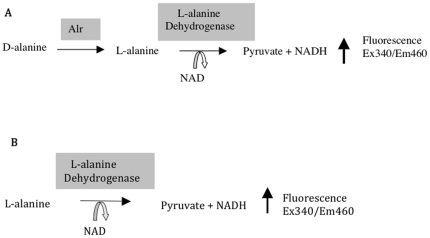
HTS assays for screening alanine racemase-specific inhibitors. (A) Coupled alanine racemase assay. (B) L-alanine dehydrogenase coupling enzyme assay.

The two enzymatic assays developed for screening alanine racemase-specific inhibitors are shown in Figure 2. The first assay is based on the Esaki and Walsh reaction where the conversion of D-alanine to L-alanine by alanine racemase is coupled to the deamination of L-alanine to pyruvate by the NAD-dependent L-alanine dehydrogenase (Figure 2A) [33]. The formation of NADH from this reaction can be quantified fluorometrically following excitation at 340 nm and emission at 460 nm. This coupled reaction offers a convenient, cost-effective and straightforward means to screen for alanine racemase inhibitors in a high-throughput setting. One drawback of this assay however, is its inability to discriminate inhibitors of the coupling enzyme. Since the latter would result in high rates of false positives, it was necessary to develop a second assay to identify inhibitors of the coupling enzyme. Shown in Figure 2B, this assay essentially consists of the second half of the coupled reaction and directly measures the deamination of L-alanine to pyruvate.

**Figure 3:**
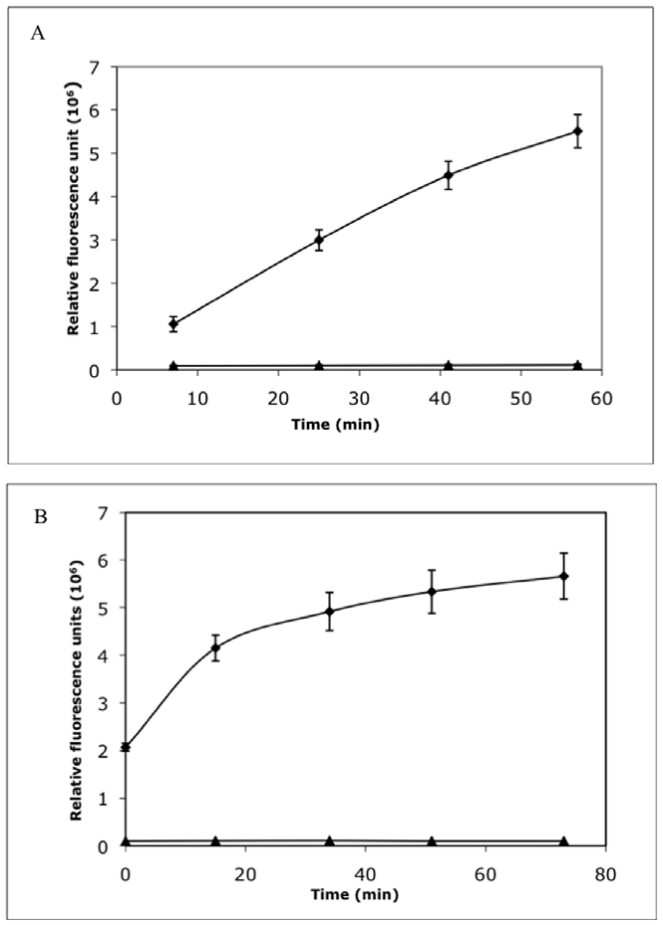
Kinetics of alanine racemase and L-alanine dehydrogenase activities in HTS format. (A) When converted to 384-well plate format, the alanine racemase reaction gives a linear increase in fluorescence over a 40-minute period (⧫) as compared to the background control without substrate (▴). (B) The L-alanine dehydrogenase reaction gives a linear increase in fluorescence over a 20-minute period (▪) as compared to the background control without substrate (⧫).

The two assays were adapted from a 1-ml cuvette format to a 40-µl 384-well plate format and optimized for HTS. Varying concentrations of substrates, enzymes and NAD co-factor were tested to obtain conditions that would be suitable for HTS. The optimized racemase assay yielded a reaction that proceeded linearly over a 40-minute period with a signal-to-background ratio of 10 (Figure 3A). The optimized coupled assay, yielded a reaction that proceeded linearly over a 20-minute period with a signal-to-background ratio of at least 10 (Figure 3B). For both assays, the calculated values for signal-to-background ratio and the screening window co-efficient (Z′ factor >0.7) indicated that the assays were suitable for HTS [34].

### High-throughput screening for *M. tuberculosis* alanine racemase inhibitors

**Figure 4:**
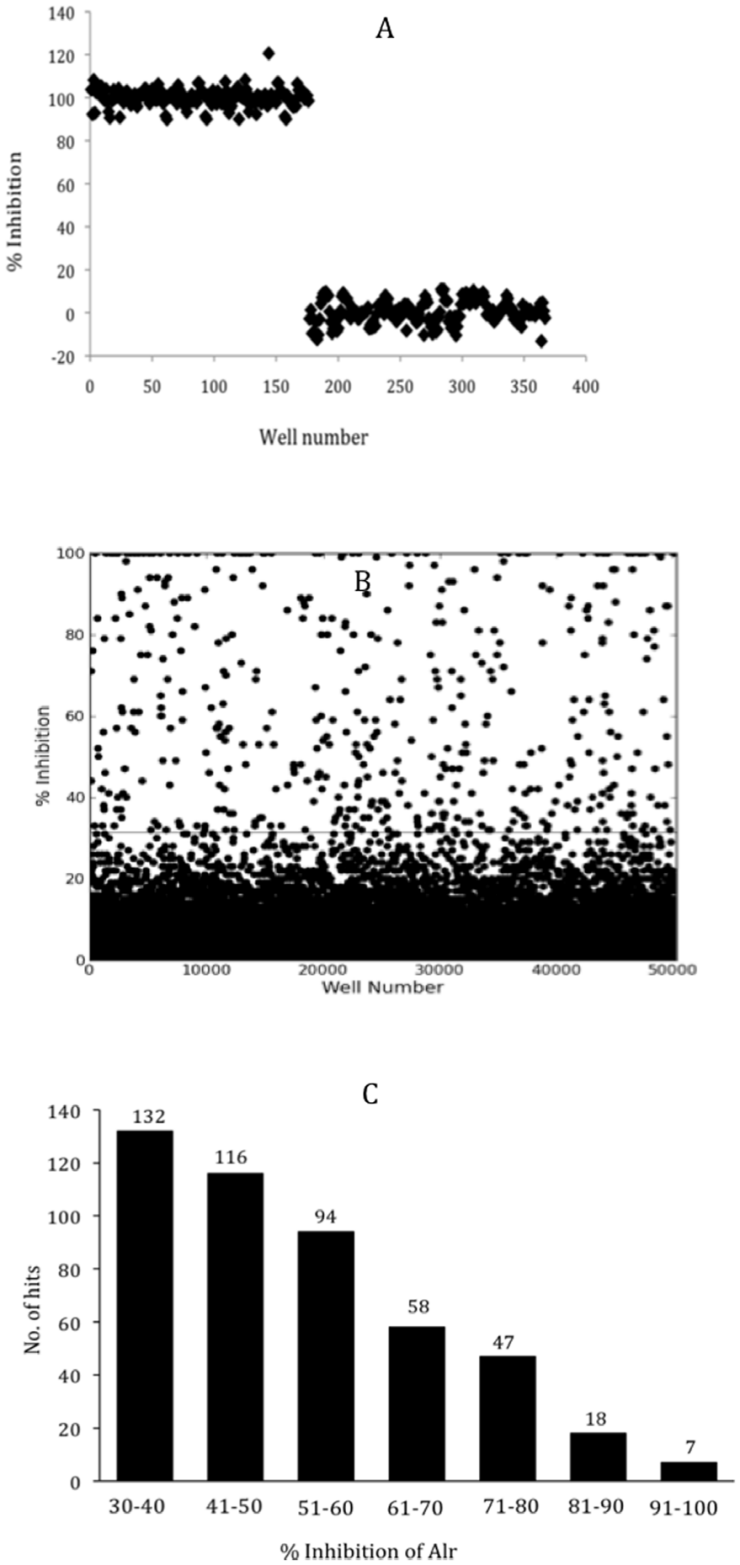
Summary of HTS outcome. (A) Results of positive and negative controls plotted for 367 representative wells of the alanine racemase assay. The positive control represents 100% inhibition (wells 1–176) and the negative control represents 0% inhibition in the presence of 1% DMSO (wells 177–367). (B) HTS data for one of the two replicates for the entire 53,000-compound screen, obtained from 138 384-well plates. The line indicates the 30% inhibition cut-off for hit selection. A similar distribution of positive and negative controls results were obtained for the coupling enzyme assay (data not shown). (C) Distribution of the 267 hits, with the hits categorized into 7 groups with respect to percent inhibition.

The two assays were used to screen 53,000 synthetic compounds and natural product extracts from 13 different libraries. The libraries were screened in duplicate at final concentrations of 12.5 µg/ml for synthetic compounds and 37.5 µg/ml for natural products. Reaction mixtures containing the enzymes were pre-incubated with each test substance in 384-well plates. Positive and negative controls (without inhibitor and substrate, respectively) were included in each plate. After a 30-minute pre-incubation, substrates were added and fluorescence intensity was read. The percent inhibition for each test substance was calculated with respect to the controls and the plots are shown in Figure 4. With respect to the controls (Figure 4A), the majority of the 53,000 compounds did not show inhibition (Figure 4B). Compounds showing greater than 30% inhibition in the alanine racemase assay and less than 10% inhibition in the coupling enzyme assay were designated ‘hits’. Based on these criteria, 472 compounds were identified as hits for a hit-rate of 0.9%. All the hits were from synthetic compound libraries. The hits were ranked for potency based on percent inhibition of alanine racemase. As shown in Figure 4C, there were 25 strong (>80%), 199 intermediate (51–80%) and 248 weak (30–50%) alanine racemase inhibitors.

### Further selection criteria for hits and determination of alanine racemase IC_50_

All the HTS hits were ranked based on structure-based calculated properties, giving priority to those with low molecular weights, high inhibition potency, lower numbers of rotatable bonds, hydrogen bond donors and acceptors, lower polar surface area, and lack of highly reactive groups [36]. Based on this analysis, 167 hits were cherry-picked and retested in a 5-fold dilution series (5.7, 1.1, 0.229, 0.046, 0.009 µg/ml). Sixteen of these compounds specifically inhibited alanine racemase in a dose-dependent manner with more than 50% inhibition at the highest dose. Table 1 shows the chemical structures of the 16 new alanine racemase inhibitors, which include two hydrazides (L2-1 and 2), two pyrimidine carboxamides (L2-5 and 6), three thioamides (L2-7, 9, and 12), one each of hydroxamic acid (L2-3), thiopyridine (L2-4), pyridine ester (L2-10), oxadiazole N-oxide (L2-18), carbamodithioate (L2-11), isothiazolopyridione (L2-13), benzopyranone oxime (L2-14), alkoxy phenol (L2-15), and quinolinedione (L2-16). These compounds were purchased and their enzyme IC_50_ values were determined. The results, summarized in Table 1, show that the IC_50_ values ranged from 1 to 13 µM, with the pyridine ester (L2-10) showing the most potent activity.

**Table 1:** Chemical properties and activities of new alanine racemase inhibitors and cycloserine*.

Hits	Inhibitor	MW	IC_50_µM	1Mtb	2MIC µM	3TC_50_µM	4T_i_	*Ki*mM
L2-01	N′,N′,4-trimethylbenzenesulfonohydrazide	214	9.0	−	ND	ND	ND	ND
L2-02	2-N′,2-N′,7-N′,7-N′-tetramethyl-9H-fluorene-2,7-disulfonohydrazide	410	1.6	−	ND	ND	ND	ND
L2-03	N-hydroxy-2-(2-hydroxyphenoxy)acetamide	183	8.2	−	ND	ND	ND	ND
L2-04	ethyl 3-(pyridin-2-ylthio)propanoate	211	2.6	+	59	229.8	4	0.038
L2-05	N-benzyl-5-chloro-2-methylsulfonylpyrimidine-4-carboxamide	326	6.8	+	9	20	2	0.63
L2-06	5-chloro-N-(3-chloro-4-methoxyphenyl)2(methylsulfonyl)pyrimidine-4carboxamide	346	8.2	+	<4.5	18.8	4	0.76
L2-07	2-(4-methylphenyl)-1-morpholin-4-ylethanethione	235	6.5	−	ND	ND	ND	ND
L2-09	2-(4-methoxyphenyl)-1-morpholin-4-ylethanethione	251	3.3	−	ND	ND	ND	ND
L2-10	6-O-[3-chloro-4-(6-methoxycarbonylpyridine-2-carbonyl)oxyphenyl]2-O-methyl pyridine-2,6-dicarboxylate	471	1.0	+	13.6	157.2	12	0.68
L2-11	2-(pyridin-3-ylcarbamothioyl sulfanyl)acetic acid	228	13.1	−	ND	ND	ND	ND
L2-12	2-phenyl-1-piperidin-1-ylethanethione	219	6.0	+	28.6	406	14	0.08
L2-13	2-(4,6-dimethyl-3-oxo-[1], [2]thiazolo[5,4-b]pyridin-2-yl)-N-[2-(4-ethoxyphenyl)ethyl]acetamide	384	7.7	+	16.2	36.4	2	0.93
L2-14	2-(hydoxyimino)-6-methyl-2H-benzopyran-3-carboxamide	208	2.8	−	ND	ND	ND	ND
L2-15	2-(2-hydroxyphenoxy)-N-methylacetamide	181	5.7	−	ND	ND	ND	ND
L2-16	3,3-dihydroxy-1H-quinoline-2,4-dione	193	5.2	+	32.4	33.7	1	0.02
L2-18	1,1′-(2-oxido-1,2,5-oxadiazole-3,4-diyl)-bis (1-(2-thienyl))-methanone	288	4.9	−	ND	ND	ND	ND
CS	(4R)-4-amino-3-isoxazolidinone	102	58	+	65	203	3	0.086

1 antimicrobial activity against *M. tuberculosis* (+ active, − inactive);

2 MIC against *M. tuberculosis*;

3 Cytotoxicity in HeLa cells,

4 Ti = TC_50_/MIC, ND- not determined; CS- cycloserine.

*Chemical structures of inhibitors are provided in Table S1.

### Antimicrobial activity of 16 new alanine racemase inhibitors against *M. tuberculosis*

All compounds were screened for antimicrobial activity against *M. tuberculosis* H37Rv. BACTEC culture tubes were inoculated with *M. tuberculosis* H37Rv, and inhibitors were added to a final concentration of 50 and 100 µg/ml. Cycloserine and DMSO solvent were included as positive and negative controls, respectively. The results are summarized in Table 1. Of the 16 compounds tested, seven compounds (L2-04, L2-05, L2-06, L2-10, L2-12, L2-13, and L2-16) inhibited growth at both concentrations.

To corroborate these findings, we proceeded to determine the minimal inhibitory concentrations (MIC) of the 7 active compounds using the microbroth dilution method as we have previously reported [35]. The results, which are summarized in Table 1, show that the antimicrobial activities of all seven compounds were confirmed. Their MICs ranged from <1.56 to 12.5 µg/ml (<4.5 µM to 59 µM), several of which were lower than the 6.5 µg/ml (65 µM) MIC of cycloserine. To determine if the observed growth inhibition was due to inhibition of cellular alanine racemase, we measured the MICs in the presence of 5 mM D-alanine in the growth medium. While a 3-fold increase in the MIC was observed for cycloserine, no significant increase was observed for any of the seven inhibitors, suggesting that their antimicrobial activity might not due solely to inhibition of cellular alanine racemase (data not shown).

### Cytotoxicity studies of seven alanine racemase inhibitors with antimicrobial activity

We proceeded to examine the cytotoxicity of the seven active compounds in a mammalian cell line. HeLa cells were exposed to varying concentrations of the seven compounds for 48 hours. The effects of the compounds on cellular viability were determined by measuring mitochondrial activity in live cells. The TC_50_ value (compound concentration that causes 50% cell death) of each compound is shown in Table 1. The values for the seven compounds ranged from 33–406 µM while the TC_50_ of cycloserine was 203 µM. Therapeutic indices (Ti) of the compounds were calculated to gauge the suitability of the compounds as potential leads. The results, which are summarized in Table 1, indicated that with the exception of L2-16, which was cytotoxic at slightly above its MIC (Ti = 1), the remaining compounds did not show cytotoxicity at their respective MICs, suggesting their suitability as potential leads.

### Enzyme affinity (*K_i_*) of seven alanine racemase inhibitors with antimicrobial activity

Next we determined the enzyme affinity of the seven inhibitors. For *K_i_* determination, enzyme activity was measured at 15 different inhibitor concentrations (0.01 to 0.98 mM) and at five different substrate concentrations (0.5–2.5 mM). As shown in Table 1, the *K_i_* values of the seven compounds ranged from 0.038 to 0.927 mM.

### Analysis of inhibitor enzyme interaction by mass spectrometry

**Figure 5:**
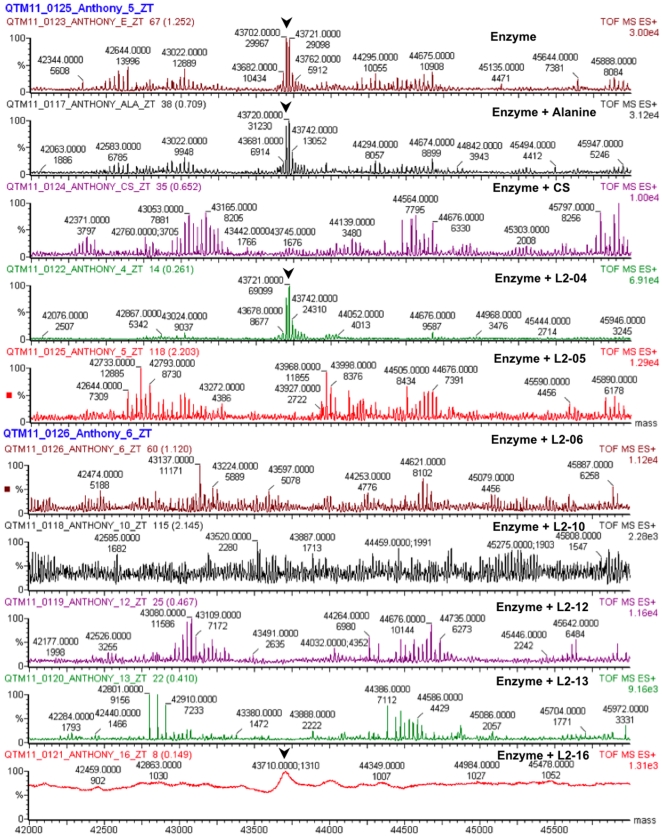
Electrospray ionization mass spectrometry analysis of enzyme-inhibitor interaction. Alanine racemase (4 µM) and inhibitors or substrate (1 mM) mixtures were analyzed by ESMS. Arrowheads indicate the peaks corresponding to monomeric alanine racemase.

Since the coupled enzyme assay does not reveal direct interaction of inhibitors with alanine racemase, we used mass spectrometry to detect if actual binding occurred and if so, the nature of the binding. Mixtures of alanine racemase with each of the seven inhibitors, cycloserine, and alanine were analyzed by ESMS and the results are shown in Figure 5. Under these conditions, the unmodified monomeric enzyme has two peaks corresponding to molecular weights 43,702 and 43,721. In the presence of alanine, L2-04, and L2-16 the peaks remain unchanged, suggesting that like the substrate, these two inhibitors likely interact with the enzyme in a reversible manner. In the presence of the irreversible inhibitor cyloserine, the monomeric peaks disappear with concomitant appearance of higher and lower molecular weight peaks, indicating irreversible modification of the enzyme. Similar results were observed with L2-05, L2-06, L2-12 and L2-13, suggesting that these inhibitors interact with the enzyme in an irreversible manner. The results for L2-10, however, were not definitive as the peak profile was ambiguous. Further studies are currently underway to elucidate the structural basis and mode of action of these inhibitors.

## Discussion

As an essential and uniquely prokaryotic enzyme, alanine racemase has long been pursued as a target for antimicrobial drug discovery. Numerous enzyme inhibitors have been identified. The vast majority, however, are suicide substrates that react with the enzyme cofactor and tend to indiscriminately inhibit other PLP-containing enzymes. In an effort to obtain more target-specific inhibitors, we performed HTS and identified 16 novel alanine racemase inhibitors that are not substrate analogs. A subset of these inhibitors blocked *M. tuberculosis* growth and showed low cytotoxicity toward mammalian cells, making them promising leads and warranting further studies into their mechanism of action.

Mass spectromentry analysis revealed that two of the inhibitors likely bind the enzyme in a reversible manner while four inhibitors are irreversible binders. It is highly unlikely that the inhibitors bind the enzyme through interaction with PLP, primarily due to a missing free amino group, requisite for external aldimine formation with the co-factor [28]. Based on the crystal structure of *M. tuberculosis* alanine racemase, there are at least three possible ways by which an inhibitor that does not engage the PLP could block enzyme function. An inhibitor could directly bind to the active site, or alternatively, it could block the formation of the active sites by blocking enzyme dimerization. All of these possibilities are currently being investigated with efforts to obtain enzyme-inhibitor co-crystals.

Some additional comments can be made concerning structure-activity relationships (SAR) among the inhibitors in Table 1; however, given the relatively small data set such SAR proposals should be considered preliminary at this time. In any event, among the three thioamides (L2-7, L2-9, and L2-12), whereas they all had similar alanine racemase enzyme IC_50_s, only compound L2-12 had significant activity against *M. tuberculosis* in cell culture. This would suggest that either a hydrogen on the *para* position of the phenyl ring is favored over a methyl or methoxy group, or a piperidine amide rather than a morpholine amide is necessary for MICs, or both. Note that another thioketo derivative, namely the carbamothioyl analog L2-11, had activity against alanine racemase; however, it did not show significant *M. tuberculosis* activity. With regards to the two pyrimidine carboxamide derivatives L2-05 and L2-06, while both had similar activity against the enzyme, it would appear that more potent MIC activity is obtained with L2-06 suggesting that a phenyl amide is preferred over a benzyl amide for best cell culture activity. Inhibitors L2-01 and L2-02 are both sulfonylhydrazides, with L2-02 showing significantly improved alanine racemase enzyme inhibition over L2-01 suggesting that either the larger size of L2-02 is allowing for additional hydrophobic (possible π-stacking) interactions relative to L2-01, or else the additional sulfonylhydrazide in L2-02 is responsible for another favorable interaction with alanine racemase. The phenols L2-03 and L2-15 may share a similar binding site in the enzyme, and, if so, this would suggest that the methyl amide can be replaced with a hydroxamide without significant loss of enzyme activity. As more data is obtained on newer analogs of compounds in Table 1, these preliminary SAR suggestions will likely be both refined and expanded.

The antimycobacterial activities noted for seven of the inhibitors are particularly encouraging, and highlight the feasibility of obtaining, by HTS, not only structurally new alanine racemase inhibitors, but also compounds that are able to cross the mycobacterial cell wall. The seven inhibitors show good MICs, which range from <4.5 µM to 59 µM. However, preliminary experiments with exogenously added D-alanine suggest that the observed MICs may not be due solely to inhibition of alanine racemase, and that additional cellular targets for these inhibitors likely exist.

Cytotoxicity, which is often used as a criterion to select compounds for lead development revealed that six inhibitors, with the exception of L2-16, were not cytotoxic at their MIC concentrations. The thioamide L2-12, though not cytotoxic, is generally not considered a good therapeutic lead due to its potential oxidation into toxic reactive species *in vivo*. This leaves five new inhibitors as possible leads for further studies.

### Conclusion

Alanine racemase has often been proposed as a target for drug design in antimicrobial development. Though several attempts have been made to rationally design enzyme inhibitors, none has produced candidates superior to cycloserine, the toxic TB drug that interacts with the PLP cofactor and blocks enzyme activity in a non-specific manner. To identify inhibitors that do not share features of cycloserine or any other previously known alanine racemase inhibitors, we performed HTS of chemical compound libraries. We identified several novel non-substrate alanine racemase inhibitors. These belong to different chemical classes, including hydrazide, hydroxamic acid, thiopyridine, pyrimidine carboxamide, thioamide, pyridine ester, oxadiazole N-oxide, carbamodithioate, isothiazolopyridione, benzopyranone oxime, alkoxy phenol, and quinolinedione. Seven of these compounds inhibited *M. tuberculosis* growth, several of which were minimally cytotoxic. Collectively, these results demonstrate the feasibility of using HTS to obtain novel alanine racemase inhibitors that are potentially useful for development as anti-TB agents.

## Supporting Information

Table S1 Chemical properties and activities of new alanine racemase inhibitors and cycloserine.
(DOC)
