# Supplementary material for: New Classes of Alanine Racemase Inhibitors Identified by High-Throughput Screening Show Antimicrobial Activity against Mycobacterium tuberculosis
Source: PLoS One. 2011 May 26;6(5):e20374. doi: 10.1371/journal.pone.0020374 (PMC3102704; doi:10.1371/journal.pone.0020374)
Supplement: Table S1 — Chemical properties and activities of new alanine racemase inhibitors and cycloserine. (DOC) [file pone.0020374.s001.doc]

Table 1. Chemical properties and activities of new alanine racemase inhibitors and cycloserine.

| **Hits** | **Chemical name** | **MW** | **IC_50_**  **μM** | **^1^Mtb** | **^2^MIC μM** | **^3^TC_50_**  **μM** | **^4^T_i_** | ***Ki***  **mM** | **Inhibitor** |
| --- | --- | --- | --- | --- | --- | --- | --- | --- | --- |
| L2-01 | N',N',4-trimethylbenzenesulfonohydrazide | 214 | 9.0 | - | ND | ND | ND | ND | 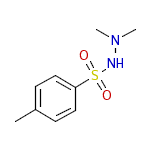 |
| L2-02 | 2-N',2-N',7-N',7-N'-tetramethyl-9H-fluorene-2,7-disulfonohydrazide | 410 | 1.6 | - | ND | ND | ND | ND | 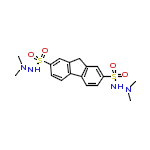 |
| L2-03 | N-hydroxy-2-(2-hydroxyphenoxy)acetamide | 183 | 8.2 | - | ND | ND | ND | ND | 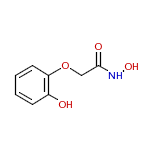 |
| L2-04 | ethyl 3-(pyridin-2-ylthio)propanoate | 211 | 2.6 | + | 59 | 229.8 | 4 | 0.038 | 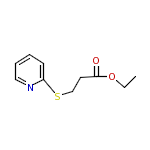 |
| L2-05 | N-benzyl-5-chloro-2-methylsulfonylpyrimidine-4-carboxamide | 326 | 6.8 | + | 9 | 20 | 2 | 0.63 | 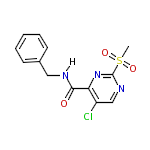 |
| L2-06 | 5-chloro-N-(3-chloro-4-methoxyphe  nyl)2(methylsulfonyl)pyrimidine-4carboxamide | 346 | 8.2 | + | <4.5 | 18.8 | 4 | 0.76 | 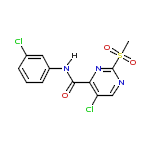 |
| L2-07 | 2-(4-methylphenyl)-1-morpholin-4-ylethanethione | 235 | 6.5 | - | ND | ND | ND | ND | 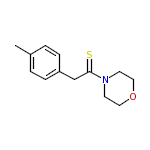 |
| L2-09 | 2-(4-methoxyphenyl)-1-morpholin-4-ylethanethione | 251 | 3.3 | - | ND | ND | ND | ND | 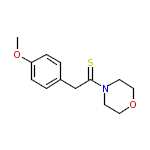 |

### Table 1. Continued.

| **Hits** | **Chemical name** | **MW** | **IC_50_**  **μM** | **Mtb** | **MIC μM** | **TC_50_**  **μM** | **^4^T_i_** | ***Ki***  **mM** | **Inhibitor** |
| --- | --- | --- | --- | --- | --- | --- | --- | --- | --- |
| L2-10 | 6-O-[3-chloro-4-(6-methoxycarbonyl  pyridine-2-carbonyl)oxyphenyl]  2-O-methyl pyridine-2,6-dicarboxylate | 471 | 1.0 | + | 13.6 | 157.2 | 12 | 0.68 | 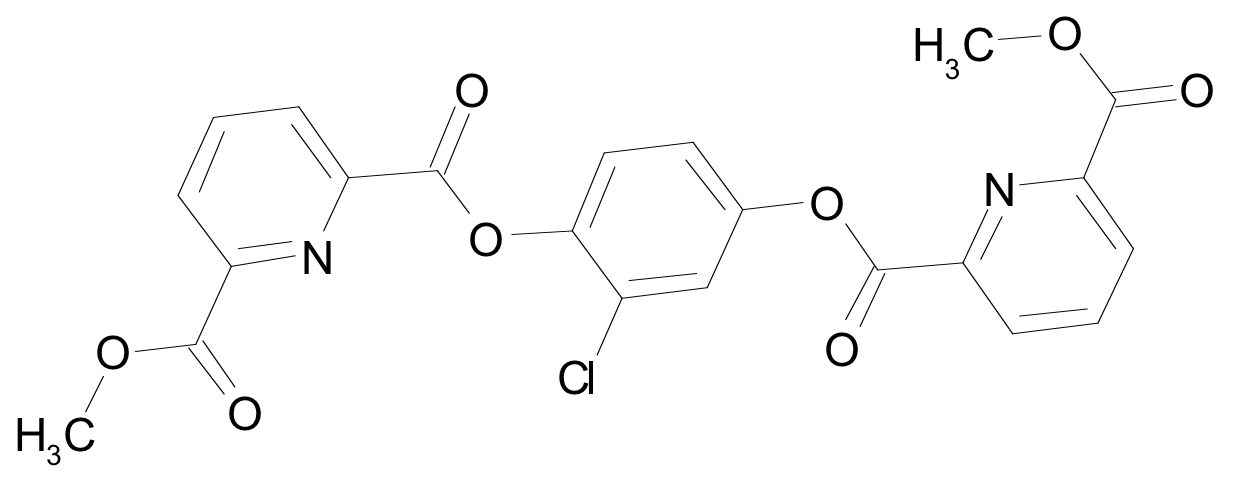 |
| L2-11 | 2-(pyridin-3-ylcarbamothioyl  sulfanyl)acetic acid | 228 | 13.1 | - | ND | ND | ND | ND | 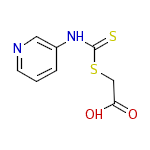 |
| L2-12 | 2-phenyl-1-piperidin-1-ylethanethione | 219 | 6.0 | + | 28.6 | 406 | 14 | 0.08 | 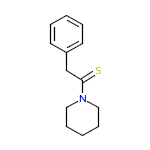 |
| L2-13 | 2-(4,6-dimethyl-3-oxo-[1,2]thiazolo[5,  4-b]pyridin-2-yl)-N-  [2-(4-ethoxyphenyl)  ethyl]acetamide | 384 | 7.7 | + | 16.2 | 36.4 | 2 | 0.93 | 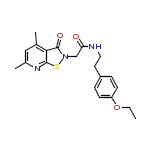 |
| L2-14 | 2-(hydoxyimino)-6-methyl-  2H-benzopyran-3-carboxamide | 208 | 2.8 | - | ND | ND | ND | ND | 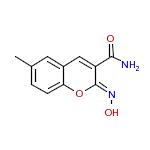 |
| L2-15 | 2-(2-hydroxyphenoxy)-N-methylacetamide | 181 | 5.7 | - | ND | ND | ND | ND | 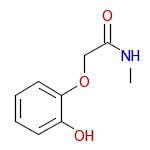 |
| L2-16 | 3,3-dihydroxy-1H-quinoline-2,4-dione | 193 | 5.2 | + | 32.4 | 33.7 | 1 | 0.02 | 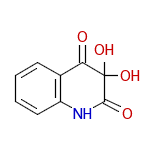 |
| L2-18 | 1,1’-(2-oxido-1,2,5-oxadiazole-  3,4-diyl)-bis (1-(2-thienyl))-  methanone | 288 | 4.9 | - | ND | ND | ND | ND |  |
| CS | (4R)-4-amino-3-isoxazolidinone | 102 | 58 | + | 65 | 203 | 3 | 0.086 | 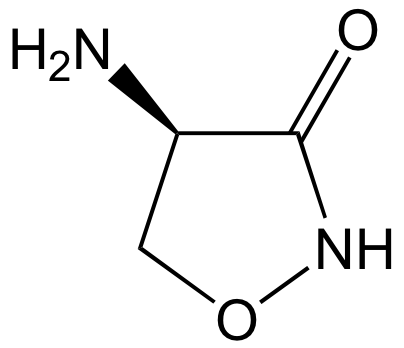 |

^1^ antimicrobial activity against *M. tuberculosis* (+ active, - inactive); ^2^MIC against *M. tuberculosis*; ^3^Cytotoxicity in HeLa cells, ^4^Ti= TC_50_/MIC, ND- not determined; CS- cycloserine
